# Restoration of Physiological Levels of Uric Acid and Ascorbic Acid Reroutes the Metabolism of Stored Red Blood Cells

**DOI:** 10.3390/metabo10060226

**Published:** 2020-05-29

**Authors:** Manon Bardyn, Jingkui Chen, Michaël Dussiot, David Crettaz, Lucas Schmid, Emmanuel Längst, Pascal Amireault, Jean-Daniel Tissot, Mario Jolicoeur, Michel Prudent

**Affiliations:** 1 Laboratoire de Recherche sur les Produits Sanguins, Transfusion Interrégionale CRS, CH-1066 Epalinges, Switzerland; Manon.Bardyn@itransfusion.ch (M.B.); David.Crettaz@itransfusion.ch (D.C.); lucas.schmid@alumni.epfl.ch (L.S.); Emmanuel.Laengst@itransfusion.ch (E.L.); 2 Département de Génie Chimique, École Polytechnique de Montréal, P.O. Box 6079, Centre-Ville Station, Montréal, QC H3C 3A7, Canada; jingkui.chen@polymtl.ca (J.C.); mario.jolicoeur@polymtl.ca (M.J.); 3 INSERM UMR 1163, Laboratory of Cellular and Molecular Mechanisms of Hematological Disorders and Therapeutical Implications, INTS, Université de Paris, 75015 Paris, France; michael.dussiot@gmail.com (M.D.); pamireault@ints.fr (P.A.); 4 Laboratoire d’Excellence GR-Ex, 75015 Paris, France; 5 Centre de Transfusion Sanguine, Faculté de Biologie et de Médicine, Université de Lausanne, CH-1011 Lausanne, Switzerland; doyen.fbm@unil.ch; 6 INSERM UMR_S1134, BIGR, Université de Paris, 75739 Paris, France

**Keywords:** red blood cell, blood storage, transfusion, metabolomics, antioxidant, uric acid, ascorbic acid

## Abstract

After blood donation, the red blood cells (RBCs) for transfusion are generally isolated by centrifugation and then filtrated and supplemented with additive solution. The consecutive changes of the extracellular environment participate to the occurrence of storage lesions. In this study, the hypothesis is that restoring physiological levels of uric and ascorbic acids (major plasmatic antioxidants) might correct metabolism defects and protect RBCs from the very beginning of the storage period, to maintain their quality. Leukoreduced CPD-SAGM RBC concentrates were supplemented with 416 µM uric acid and 114 µM ascorbic acid and stored during six weeks at 4 °C. Different markers, i.e., haematological parameters, metabolism, sensitivity to oxidative stress, morphology and haemolysis were analyzed. Quantitative metabolomic analysis of targeted intracellular metabolites demonstrated a direct modification of several metabolite levels following antioxidant supplementation. No significant differences were observed for the other markers. In conclusion, the results obtained show that uric and ascorbic acids supplementation partially prevented the metabolic shift triggered by plasma depletion that occurs during the RBC concentrate preparation. The treatment directly and indirectly sustains the antioxidant protective system of the stored RBCs.

## 1. Introduction

The stress that red blood cells (RBCs) accumulate during their storage (up to six weeks at 4 °C) triggers damages that can impair transfusion and are suspected to be associated with deleterious side effects, that could affect the patient’s clinical outcome [1,2,3,4,5]. The physicochemical changes that packed RBCs accumulate during their storage are first reversible (both in vitro and in vivo). For example, intracellular adenosine triphosphate (ATP) and 2,3-diphosphoglycerate (2,3-DPG) levels recover rapidly in the hours following transfusion [6]. However, when storage duration is extended beyond four weeks, the RBC energetic, protective and repair systems are so profoundly affected that irreversible lesions begin to accumulate [7,8,9,10]. Nowadays, several discussions are still ongoing in the transfusion community concerning the potential deleterious effect of transfusing “old” blood [11,12,13]. Nevertheless, behind the debate about the product safety, one should also think about its quality. It undoubtedly declines with storage duration because of the impairment of several biochemical and biological functions, impacting the transfusion efficiency. It thus feeds the debate in transfusion praxis related to the age of the products [5,12,14].

The roots of the RBC storage lesions are the accumulation of oxygen within the RBC concentrates (RCCs) that contributes to oxidative stress, as well as the ex vivo conditions that differ from physiological ones. It leads to remodelling of the cell metabolism (e.g., reduction in temperature slows down most physiological processes). Furthermore, RBCs are stored in additive solution (e.g., saline–adenine–glucose–mannitol (SAGM)), within the quasi-closed environment of a gas-permeable plastic bag. During RBC storage, essential metabolites are consumed, while waste products accumulate inside and outside the cells. The slightly acidic pH of additive solutions on combination to lactate accumulation (glycolysis end-product) slow down the enzymatic activity [15]. In addition, different salvage mechanisms triggered by the accumulation of storage lesions participate to metabolism remodelling. Instead of a constant decay, three phases are observed with distinct metabolite signatures during storage at 4 °C with transitions at days 10 and 18, as described by Bordbar et al. [16].

During labile blood product preparation, plasma is removed and almost entirely replaced by the additive solution [17], which creates a new equilibrium between the intra- and extra-cellular compartments. For example, it is likely that the uric acid (UA)-rich intracellular compartment starts leaking in the UA-poor supernatant in the hours following RCC preparation [16]. Maximum concentration of extracellular UA is reached after 7 to 10 days of storage and is followed by a decay [18]. The loss of UA might in theory: (1) induce a metabolic shift (UA being a purine metabolic pathway end-product), as well as (2) reduce the extra- and/or intra-cellular antioxidant defences. Indeed, UA is an endogenous antioxidant able to reduce a large range of reactive oxygen species (ROS) and more particularly the singlet O_2_ molecules and free radicals [19,20]. UA represents approximatively 60% of the plasma antioxidant capacity [21], with a normal serum level comprised between 120 and 420 µM, close to its solubility limit [22]. During RBC storage, the concentration of plasma UA was shown to be a potential hallmark of storage. Indeed, RBCs from donors having an elevated plasmatic UA concentration store better [23,24], with reduced indicators of oxidative stress, intracellular Ca^2+^ accumulation and spheroechinocytosis.

The aim of the present study was to evaluate RBC metabolism modulation along storage. In particular, the strategy consisted in restoring, in the blood bag, physiological levels of UA, an endogenous protective molecule. Ascorbic acid (AA) was reported to have a limited effect on RBC storage [25] and was added here to prevent the pro-oxidant properties of UA [26]. Indeed, it has been shown that, paradoxically, UA plays a role of antioxidant in hydrophilic environments, whereas it can become pro-oxidant for lipids and cell membranes [27,28].

## 2. Results

### 2.1. Evolution of Red Blood Cell Parameters

**Figure A1:**
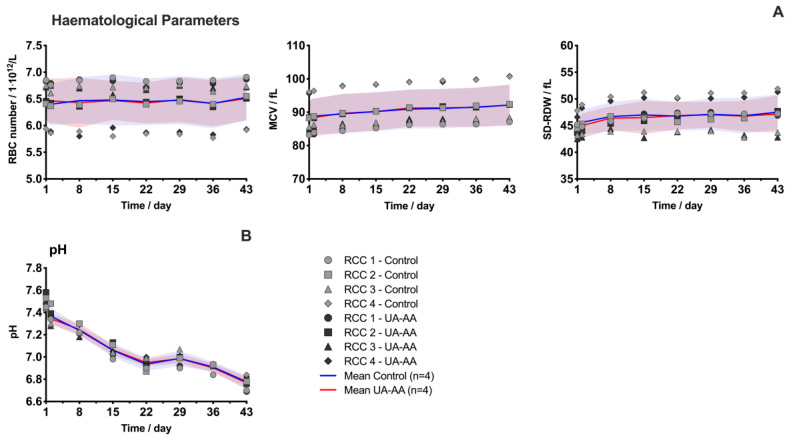
Evolution of haematological parameters and pH in red blood cell concentrates (RCCs) supplemented with uric and ascorbic acids (UA-AA) or control. (**A**) Haematological data: red blood cell (RBC) count, mean corpuscular volume (MCV) and size variation of the erythrocytes around the mean (SD-RDW) measured with Sysmex analyzer; (**B**) Evolution of the pH in the RCCs. Individual (symbols) and mean (lines) values for the four RCCs are presented ± standard deviation (shaded areas).

The haematological parameters evolved similarly for both the control and UA-AA units (Figure A1A). In all eight RCCs, cell concentration was constant, the mean corpuscular volume (MCV) increased linearly and the anisocytosis (reflected by the size variation of the erythrocytes around the mean, i.e., standard deviation of the red blood cell distribution width (SD-RDW)) became more pronounced with storage time. The pH before treatment was in average of 7.39 ± 0.05 and increased to 7.47 ± 0.04 and 7.50 ± 0.06 for the control and UA-AA units, respectively after the split and the addition of NH_3_ UA solubilisation. During storage, the pH slightly decreased because of extracellular lactate accumulation (Figure A1B).

**Figure 1:**
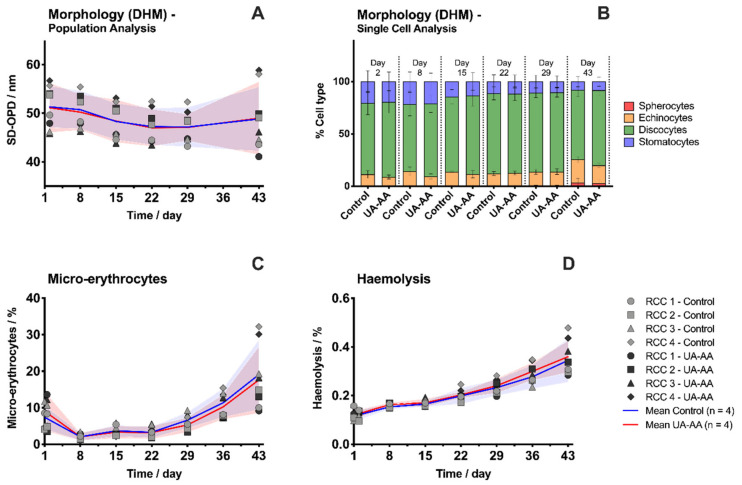
Evolution of the morphology and haemolysis in the red blood cell concentrates (RCCs) Supplemented with uric and ascorbic acids (UA-AA) or control. Morphology analysis with digital holographic microscope (DHM): (**A**) Population analysis with standard deviation of the optical path difference parameter (SD-OPD); and (**B**) single-cell analysis with CellProfiler and CellProfiler Analyst (CPA); (**C**) percentage of small cells determined with the AMNIS imaging flow cytometer; (**D**) percentage of haemolysis in the blood bag determined using the Harboe spectrophotometric method. Individual (symbols) and mean (lines) values for the four RCCs are presented ± standard deviation (shaded areas).

RBC morphology analyzed by digital holographic microscopy (DHM) was not improved by the antioxidant treatment. The cell shape integrity was maintained beyond four weeks with a stable standard deviation of the optical path difference parameter (SD-OPD, Figure 1A), which is in correlation with the amount of spherocytes (Figure 1B). After six weeks, discocytic RBCs still represented 66.4 ± 6.5% (control) and 71.8 ± 5.1% (UA-AA) of the population, in favor of the supplemented RCCs. From day 29, the number of RBCs, with irreversibly changed shapes (i.e., late stage echinocytes and spherocytes), started to accumulate.

Quantification of RBCs with reduced surface area by imaging flow cytometry confirmed the latter observations (Figure 1C). The proportion of “storage-induced micro-erythrocytes” reached 19.1 ± 9.6% of the whole RBC population for the control and of 17.6 ± 9.1% for the UA-AA units at day 43. This parameter was recently shown to correlate with the ^51^Chromium-labeled 24 h post-transfusion recovery in mouse model and in healthy human volunteers [29].

In all RCCs, a linear increase of haemolysis was observed, followed by an exponential trend after 36 days of storage (Figure 1D). At day 43, the mean haemolysis was of 0.345 ± 0.089% for the control and of 0.360 ± 0.065% for the UA-AA units, which are within the usual levels observed for SAGM RCCs [30] and below the accepted threshold at the end of the official storage period (0.8% from the European Directive of for the Quality of Medicines and Healthcare and 1% from the US Food and Drug Administration).

Globally, the two tested conditions were equivalent in term of morphology and haemolysis. UA and AA supplementation did not improve nor worsen the RBC aging based on these markers.

### 2.2. Increase of the Antioxidant Properties

**Figure 2:**
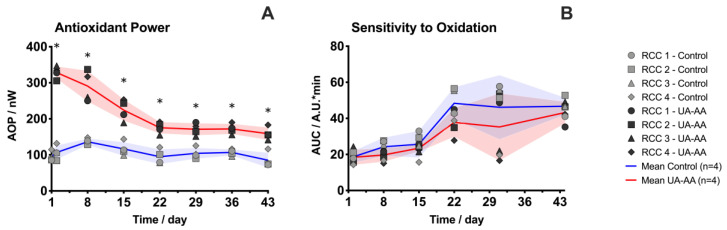
Evolution of the antioxidant power (AOP) and sensitivity to oxidation in the red blood cell concentrates (RCCs) supplemented with uric and ascorbic acids (UA-AA) or control. (**A**) AOP in supernatant quantified by electrochemical pseudo-titration with the Edel device; (**B**) RBC sensitivity to oxidation under treatment with different concentration of hydrogen peroxide (H_2_O_2_) oxidant. The generation of reactive oxygen species (ROS) was reported using the 2′,7′-dichlorofluorescin diacetate (DCFH-DA) fluorescent dye. The higher the area under the curve (AUC), the more intracellular ROS generated. Individual (symbols) and mean (lines) values for the four RCCs are presented ± standard deviation (shaded areas). * *p*-value < 0.05 two-way ANOVA.

As a direct consequence of the supplementation, the antioxidant power (AOP) quantified by electrochemical pseudo-titration was higher in UA-AA treated units (106.9 ± 19.5 nW in the control vs. 328.4 ± 17.3 nW in UA-AA RCCs at day 2, Figure 2A). Indeed, the added water-soluble antioxidants contributed directly to the AOP of supplemented RCCs. In control RCCs, the AOP increased during the first week of storage and then decreased before reaching a plateau value. Such kinetics have already been observed and shown to correlate with the extracellular UA level [18]. The observed trends in treated units (i.e., a reduction of the AOP) might be explained by either a degradation of urate (by oxidative mechanisms for instance), or an uptake of the added molecules by the RBCs. Interestingly, AOP consumption exhibited two phases with a rapid decay during the first three weeks of storage. At any time of the follow-up AOP was significantly higher in the UA-AA compared to control units.

The sensitivity to oxidative stress clearly increased (i.e., RBCs were less prone to manage oxidative imbalance) at the beginning of the second week of storage (Figure 2B). These results are in agreement with the literature where an accumulation of ROS during the first three weeks of storage was reported [31]. Thanks to the addition of exogenous antioxidants, the treated RBCs tended (although not significantly) to be more resistant to the oxidative stress artificially induced by hydrogen peroxide (H_2_O_2_) than the standard ones with a marked difference after three weeks of storage. The control and UA-AA-treated RBCs became, at day 43, respectively 2.5- and 2.3-times more sensitive to oxidation in comparison to day 2.

### 2.3. Rerouting of the Metabolism

**Figure 3:**
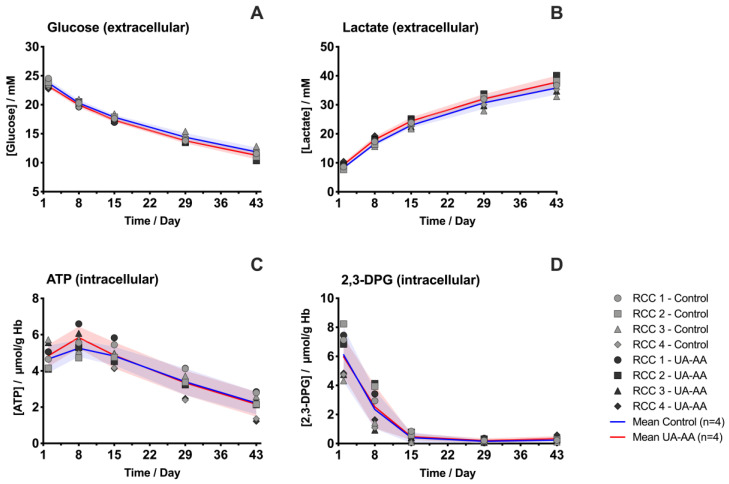
Evolution of the extracellular levels of glucose and lactate, and of the intracellular concentrations of ATP and 2,3-DPG in the red blood cell concentrates (RCCs) supplemented with uric and ascorbic acids (UA-AA) or control. The analyses were performed using commercial assay kits; (**A**) extracellular glucose level; (**B**) extracellular lactate level; (**C**) intracellular ATP level; (**D**) intracellular 2,3-DPG level. Individual (symbols) and mean (lines) values for the four RCCs are presented ± standard deviation (shaded areas).

**Figure 4:**
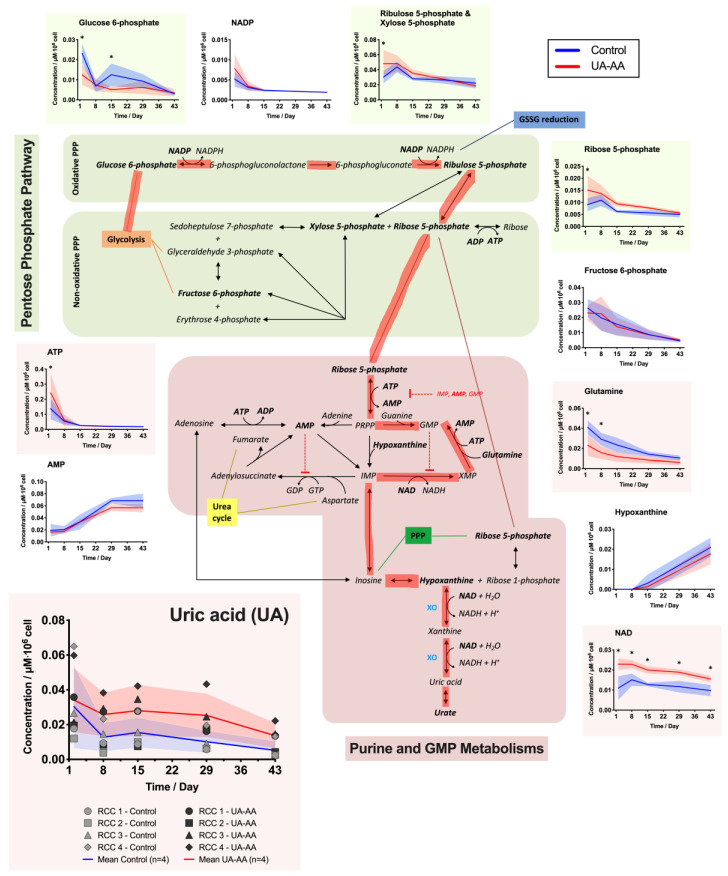
Effect of uric and ascorbic acids (UA-AA) supplementation on red blood cell (RBC) metabolism—focus on the pentose phosphate pathway (PPP), and the purine and GMP metabolisms. The graphs present the results of time-course metabolomic analysis by mass spectrometry of several intracellular RBC metabolites. In the control RBCs, the metabolism seems to be pulled in the direction (path highlighted in red) of the non-oxidative PPP and toward the purine and GMP metabolism, probably as a result of the depletion of the intracellular UA pool. Individual (symbols) and mean (lines) values for the four RCCs are presented ± standard deviation (shaded areas). * *p*-value < 0.05 two-way ANOVA.

The rates of extracellular glucose consumption and lactate accumulation were higher at the beginning of the storage, suggesting a more active glycolysis [16,30,32] (Figure 3A,B). The antioxidant treatment did not impact these kinetics. Similarly, the intracellular 2,3-DPG was rapidly depleted (within two weeks) in the control as well as under UA-AA treatment (Figure 3D). In contrast, differences were observed for the intracellular ATP levels, that were higher in treated RBCs at the beginning of the storage (Figure 3C and Figure 4).

**Figure A2:**
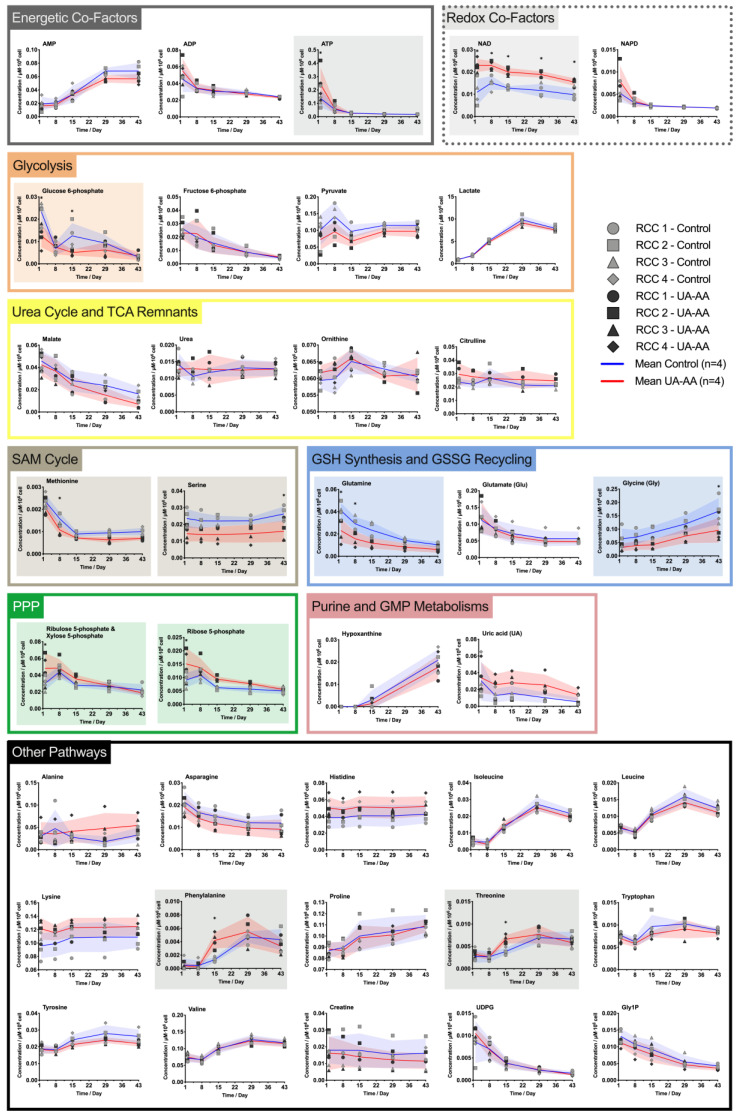
Effect of uric and ascorbic acids (UA-AA) supplementation on red blood cell (RBC) metabolism. Quantitative data of the time-course metabolomic analysis by HPLC-MS for the control RBCs stored in standard conditions (light grey lines) vs. the RBCs supplemented with UA-AA (dark grey lines). Individual (symbols) and mean (lines) values for the four RCCs are presented ± standard deviation (shaded areas). * *p*-value < 0.05 2way ANOVA.

Although the evolutions on the glucose intake and lactate release were equivalent in both conditions, the data on antioxidant properties and ATP suggest subtle changes in the metabolic pathways. The metabolomic analysis indicates that the treatment impacted cell metabolism (data of all analyzed metabolites available in Figure A2).

UA-AA supplementation seemed to reduce loss of intracellular UA. Indeed, UA was rapidly depleted during the first week in control RBCs (as observed previously [16]), but was maintained in treated cells. The difference was stable from day 2 to day 29 and decreased at the end of the storage.

**Figure 5:**
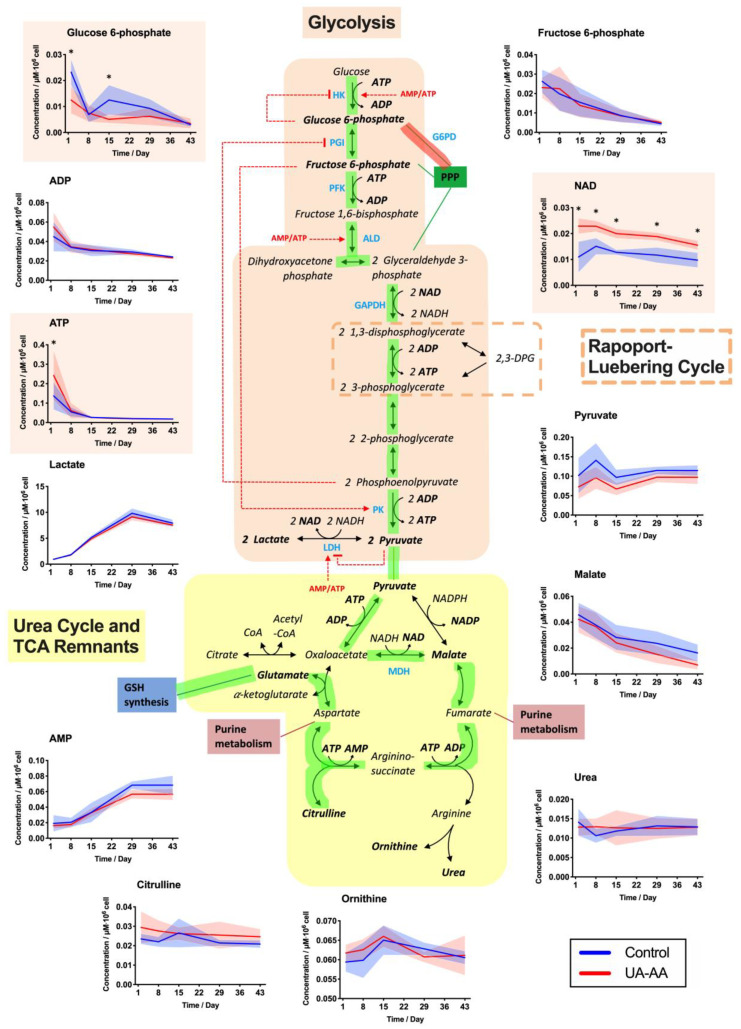
Effect of uric and ascorbic acids (UA-AA) supplementation on red blood cell (RBC) metabolism—focus on the anaerobic glycolysis, and the urea and remnants of the tricarboxylic acid cycle (TCA, or Krebs Cycle). The graphs present the results of time-course metabolomics analysis by mass spectrometry of several intracellular RBC metabolites. In the UA-AA-treated RBCs the glycolysis is favored (path highlighted in green). Mean values for the four RCCs are presented ± standard deviation (shaded areas). * *p*-value < 0.05 two-way ANOVA.

Concentrations of some energy metabolites were significantly modified (i.e., ATP and nicotinamide adenine dinucleotide (NAD)). NAD, a co-factor used in purine metabolism (Figure 4) and glycolysis (Figure 5), was lower since the beginning of the storage in UA-AA supplemented RCCs compared to control; the marked difference remained stable over storage. ATP was higher in UA-AA only during the first week and then reached the same value as in control. Of note, the increase of ATP level during the first week followed by a steady decrease observed using the enzymatic assay (Figure 3C), was not confirmed by mass spectrometry analysis, such discrepancies have already been observed in the past [33]. Finally, pyruvate (that can produce lactate or enter the urea cycle and TCA remnants) was found higher in the control (not significantly, Figure 5). Noteworthily, ribose 5-phosphate (pentose phosphate pathway, Figure 4) that is produced from glucose 6-phosphate and which enters the purine metabolism was higher in the UA-AA. This was again particularly visible at the beginning of storage right after the supplementation. An equivalent observation was made for the couple ribulose 5-phosphate/xylose 5-phosphate. On the opposite, glucose 6-phosphate was lower in the UA-AA RCCs.

**Figure 6:**
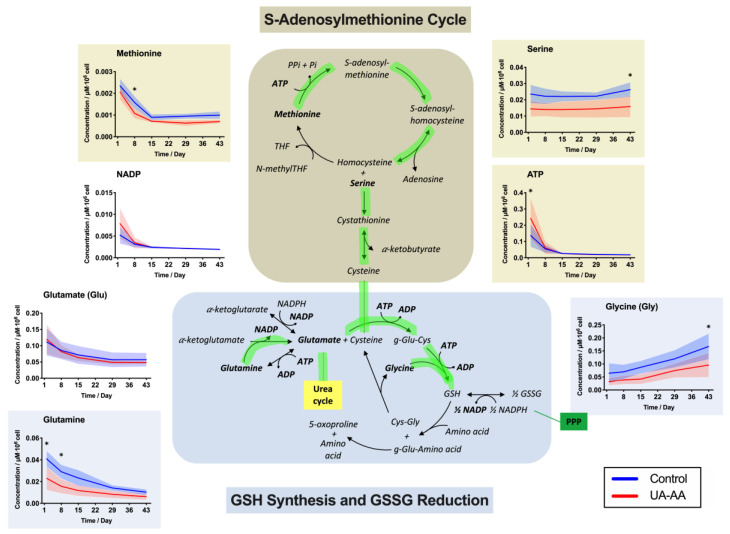
Effect of uric and ascorbic acids (UA-AA) supplementation on red blood cell (RBC) metabolism—focus on the S-adenosylmethionine cycle (SAM), and the reduced glutathione (GSH) synthesis and oxidized glutathione (GSSG) reduction pathways. The graphs present the results of time-course metabolomics analysis by mass spectrometry of several intracellular RBC metabolites. In the UA-AA-treated RBCs, the de novo synthesis of GSH seems to be favored (path highlighted in green). Mean values for the four RCCs are presented ± standard deviation (shaded areas). * *p*-value < 0.05 two-way ANOVA.

Concentration of amino-acids (i.e., methionine, serine, glutamine, glycine, phenylalanine and threonine) were significantly modified by the storage condition too. They are used, amongst others, in the S-adenosylmethionine (SAM) cycle, the de novo synthesis of reduced glutathione (GSH) and in the oxidized glutathione (GSSG) reduction pathways (Figure 6). Serine, glutamine, glycine and methionine were higher in control throughout the storage. Concentrations of phenylalanine and threonine were higher in treated RBCs at day 15. The differences were observed from day one and mainly conserved throughout the storage.

## 3. Discussion

### 3.1. Tackle the Effect of Plasma Dilution

In the present study, UA and AA molecules were added with the dual-objective to partially restore the physiological conditions impacted by the blood product preparation and to protect the RBCs against storage-related lesions.

In a general way, the UA-AA treatment modified the RBC metabolism since the beginning of the storage as shown by metabolomic analysis. Two concomitant mechanisms can explain the observed variations. First, the addition of UA at physiological concentrations counterbalances or at least limits the out-take of intracellular UA (as clearly shown in Figure 4) and the consequent metabolic dysregulation. Secondly, the addition of antioxidants preventing, at least partially, the metabolic response and modifications due to the accumulation of oxidative stress (Figure 2). UA-AA addition also improved slightly the sensitivity of RBCs to oxidation.

The changes of morphology, and the haemolysis of the stored RBCs were equivalent in control and in supplemented RCCs with UA-AA. It could be explained by the dosage (rather than of the efficacy) of the antioxidants. The specificity and sensitivity of the quality markers used could also be limited, failing to demonstrate an improvement of the storage conditions.

### 3.2. Metabolism Rerouting

Most of the time, the biggest differences in metabolite concentrations were observed at the beginning of the storage. The observations made suggest an immediate impact of the supplementation of the additive solution with UA and AA and could indicate a rerouting of the RBC metabolism. It is important to notice that the pH were identical in both conditions (Figure A1B). Therefore, the changes observed here were not triggered by the pH and were related to the added molecules.

The lower levels of methionine, serine, as well as of the GSH precursors glutamine and glycine in the UA-AA samples suggest that the treatment helped maintaining the de novo synthesis of GSH (Figure 6, pathway highlighted in green). Glutathione levels were unfortunately not monitored here. Methionine could be used by the SAM cycle to produce homocysteine, which then reacts with serine to produce cysteine. The decrease in glutamine and glycine levels tend to corroborate this hypothesis, as well as the increased consumption of pyruvate by the urea cycle and remnant of the tricarboxylic acid cycle (TCA, or Krebs cycle), that could be linked to an increase of glutamate (another GSH precursor).

A more active GSH synthesis in treated RBCs could be explained by the fact that these cells have more energy available (ATP). Indeed, the reduction of the oxidative stress by the antioxidant supplementation could limit the metabolism rerouting triggered by RBC salvage mechanisms, i.e., the shift from glycolysis toward the non-oxidative PPP that produces reduced nicotinamide adenine dinucleotide phosphate (NADPH). Such phenomena were normally shown to occur during storage in the second metabolic phase as a consequence of the oxidation of redox sensitive amino acids in the active site pocket of the glyceraldehyde 3-phosphate dehydrogenase (GAPDH) enzyme [34]. It can also be expected that less NADPH would be consumed for GSSG recycling into GSH if the oxidative burden is reduced by antioxidant supplementation provided by uric and ascorbic acids.

In conventional RCCs (control), the evolution of the metabolism suggests that the metabolic fluxes are shifted toward the oxidative PPP and then in the direction of the purine pathway, as a compensatory mechanism driven by the UA depletion which is a metabolic end-product (Figure 4, pathways highlighted in red). Such behavior is in agreement with lower levels of ribose 5-phosphate (R5P; and its isomers) and higher levels of glutamine observed in the control samples along the storage. After UA compensation, the flux through these pathways seems to slow down. Indeed, the urate level remains higher in RBCs because of the supplementation; and NAD is less consumed (or produced). These different variations indicate a rerouting in favor of the glucose consumption via the glycolysis (Figure 5, path highlighted in green) instead of feeding the pentose phosphate and the purine salvage pathways.

As for the difference in NAD concentrations, they could come from this metabolic rerouting if we assume that the RBCs can convert hypoxanthine and xanthine to UA. The hypothesis of a reaction catalyzed by the xanthine oxidase (XO) has to be confirmed since the presence of this enzyme has not been reported in RBCs as far as we know. XO catalyzes the following Reactions (1) and (2):

(1) $$\mathrm{Hypoxanthine}+{\mathrm{NAD}}^{+}+H_{2}O+O_{2}⇄\mathrm{Xanthine}\;\mathrm{oxidase}+\mathrm{NADH}+H_{2}O_{2}$$

(2) $$\mathrm{Xanthine}\;\mathrm{oxidase}+{\mathrm{NAD}}^{+}+H_{2}O+O_{2}⇄\mathrm{UA}+\mathrm{NADH}+H_{2}O_{2}$$

To validate this hypothesis, it would be necessary to investigate if the RBCs contain active XO enzymes [35]. In the future, isotopic labelling of UA could be an interesting approach to trace this molecule and determine its fate.

### 3.3. Oxidative Stress

Oxidative stress results from the imbalance between oxidants and antioxidants. In stored RBCs, the oxidative lesions which are often irreversible accumulate [7,31], due to an increase in oxygen saturation within RCC units [36], and to the reduction in the RBC antioxidant defences. The reasons are: the oxygen permeability of blood bags, the dilution of the plasma and the dysregulation of the RBC machinery controlling the amount of oxidants [37].

The blood processing itself can participate to the oxidative stress by reducing the level of intracellular antioxidants, as observed by the urate depletion. Moreover, UA depletion (control situation) could itself contribute to oxidative stress by the production of H_2_O_2_ (see Equations (1) and (2)).

Three different options can be considered to tackle such burden: (1) decrease the oxygen content, (2) sustain the redox defences, or (3) directly provide antioxidant molecules. The maintenance of the antioxidant system has been achieved by adding precursor of glutathione such as N-acetylcysteine, and various antioxidant molecules such ascorbic acid or vitamin E have been tested (see reference [5] for more details). These approaches were not able to improve the storage. The reduction of oxygen has been proposed by several groups [38,39,40,41]. In general, ATP levels were better preserved during the storage and haemolysis was reduced.

In this context, the modification of the additive solution used here contributed to improve the antioxidant properties of the RCCs. Indeed, the AOP was clearly higher than in conventional storage and the sensitivity to oxidation was lower (Figure 2). The effect was marked after three weeks of storage. At this stage of the aging, the RBC metabolism is producing hypoxanthine and xanthine that are end-products in the purine salvage pathways and could generate H_2_O_2_. It corroborates the observation made here. Therefore, we can hypothesize that the improved antioxidant properties may sustain the resistance to oxidation latter than in the conventional storage.

## 4. Materials and Methods

### 4.1. Preparation of Red Blood Cell Concentrates and Treatment

The blood used in the framework of this project was obtained from donors who gave their signed consent for the use of their blood components in research. Moreover, the project was accepted by the Institutional Review Board of Transfusion Interrégionale CRS and is in agreement with local legislation.

Four RCCs (donor’s characteristics are detailed in Table A1) were prepared using a top/bottom processing method. The whole blood (450 ± 50 mL) donated was collected in 63 mL of citrate–phosphate–dextrose (CPD) anticoagulant (CompoFlow CQ32250, Fresenius Kabi, Bad Homburg, Germany). After a holding time (7 h 26 min for RCC 1, 5 h 49 min for RCC 2, 6 h 38 min for RCC 3, 7 h 09 min for RCC 4) at room temperature (22 ± 2 °C), the bags were centrifuged (5047× *g* for 13 min at 22 °C on a Roto Silenta 630 RS, Hettich, Tuttlingen, Germany) to separate the blood components. Then, semi-automated pressure was applied (CompoMat, Fresenius Kabi, Bad Homburg, Germany) to distribute the different fractions into sterile inter-connected bags. Finally, the RBCs were filtered to deplete the leucocytes. The RCCs were stored at 4 °C in 100 mL of SAGM additive solution.

The antioxidants were added at day 1 where the UA release was limited [18]. After being split into two subunits in sealed conventional bags, the RCCs were supplemented either with 113.7 µM of AA plus 416.3 µM of UA (Sigma-Aldrich, Steinheim, Germany) dissolved in 0.9% NaCl (Laboratorium Dr G Bischel AG, Interlaken, Switzerland) (36.9 mM of NH_3_ were added to guarantee the UA solubility), or with control 0.9% NaCl plus 36.9 mM of NH_3_.

### 4.2. Follow-Up during Storage

The RCCs were sampled after 1, 2, 8, 15, 22, 29, 36, 43 days of storage. For each point of measurement, the bags were gently mixed, and a sample of approx. 4.2 mL was collected with a sterile syringe through a sampling site. Two samples were put aside. One was directly shipped at 4 °C to Paris (France) for the quantification of the small cells (AMNIS imaging flow cytometer) and the other was kept to test the sensitivity of the RBCs to oxidation.

A Sysmex automate (KX-21N, Sysmex, Kobe, Japan) provided the haematological parameters (such as the RBC count, MCV and SD-RDW). The pH was measured with a pH-meter (Orion SA 520 pH-meter and Orion Micro 8228 PerpHecT ROSS electrode). Both measurements were done on the unprocessed RCC sample.

In parallel, a 2-mL RCC sample was centrifuged at 2000× *g* for 10 min at 4 °C. The supernatant was kept for the quantification of extracellular AOP (Edel, Ecublens, Switzerland), as well as for the determination of the haemolysis percentage (Harboe spectrophotometric method with the 3-points Allen correction [42]). Some aliquots of the supernatant were also frozen and put aside for further analyses.

The RBCs were then washed twice in 0.9% NaCl. Aliquots of the RBC pellet (haematocrit (HCT) of 0.821 ± 0.03) were snap-frozen in liquid nitrogen or resuspended in two volumes of HEPA buffer for morphology analysis by DHM.

### 4.3. Morphology Analysis Using Digital Holographic Microscopy

Before morphology analysis, RBCs were diluted in HEPA buffer and seeded in the wells at a density of 80,000 cells for 100 µL per well (3 wells/sample) in a 96-well imaging plate (BD Falcon) coated with 0.1 mg/mL poly-L-ornithine as previously described [30]. The plate was centrifuged at 140× *g*, 2 min at room temperature to speed up the sedimentation process. During image acquisition, the plate was placed in a plate incubator set at 37 °C with high humidity and 5% CO_2_.

The DHM^®^ T1000 microscope (Lyncée Tec SA, Lausanne, Switzerland) is equipped with a motorized microscope stage (Märzhäuser Wetzlar GmbH & CO. KG), an incubator system (LCI Live Cell Instrument, Seoul, South Korea), as well as Leica 20×/0.40 NA and 40×/0.75 NA objectives (Leica Microsystems GmbH, Wetzlar, Germany). Quantitative phase images (20× magnification, 4 images/well) of the RBCs were acquired. DHM is a non-invasive label-free interferometric microscopy technique which provides a quantitative measurement of the phase (or optical path length), a parameter related to the morphology and haemoglobin content of the RBC [43,44,45].

The quantitative phase images were analyzed in two ways, as previously described. First, with a single-cell analysis using CellProfiler (Broad Institute, Cambridge, MA, USA, www.cellprofiler.org, 2.1.0 rev 0c7fb94) and CellProfiler Analyst (2.0 r11710) [46], that perform supervised machine learning for the classification of the RBC according to their morphology (here: “stomatocytes”, “discocytes”, “echinocytes”, or “spherocytes”). Secondly, a population analysis (yielding a single output per image) was chosen, where the SD-OPD values were calculated. The SD-OPD parameter was previously shown to be positively correlated with the percentage of the spherocytes (and also the stomatocytes) in the sample [30].

### 4.4. Storage-Induced Micro-Erythrcocytes Quantification

The projected surface area of the RBCs was characterized using imaging flow cytometry (ImageStream X Mark II, AMNIS) as described by Roussel et al. [9]. Briefly, the RBCs were diluted at 1% HCT in a modified Krebs-albumin solution and imaged at 60× magnification. The projected surface area of focused, front view RBCs was measured post-acquisition via the IDEAS software. The distribution of the projected surface area was then plotted on frequency histograms and the storage-induced micro-erythrocytes were segregated from the “normal” ones using the nadir (local minima) of the bimodal distribution.

### 4.5. Electrochemical Antioxidant Power Measurement

The Edel technology (Edel-for-Life) is an electrochemical system where screen-printed electrodes are used as sensors for AOP quantification in liquid samples [47,48]. This amperometric test measures the current generated by the oxidation at different potential (0 to 1.2 V with a scan rate of 100 mV⋅s^−1^) of water-soluble redox active species. The recorded signal is proportional to the antioxidant concentration. Pseudo-titration is used to discriminate the biologically relevant antioxidants. The AOP, expressed in nW, is calculated by the integration of the modulated current over the potential.

### 4.6. Evaluation of Red Blood Cell Sensitivity to Oxidation

The RBCs were first washed with 0.9% NaCl (centrifugation for 10 min at 2000× *g* and 4 °C). The pelleted RBCs were then resuspended at 10% HCT in 0.9% NaCl and treated with 50 µM of 2′,7′-dichlorofluorescin diacetate (DCFH-DA, Sigma-Aldrich, Steinheim, Germany). This dye is incorporated in the cells where it is de-esterified by cytosolic esterases. The DCFH molecule becomes fluorescent (Ex/Em 485/520 nm) upon oxidation by ROS [49]. The RBCs were incubated 30 min at 37 °C under agitation to enable incorporation of the reported probe. After incubation, the samples were centrifuged (as before) to get rid of the excess of DCFH-DA. Finally, the RBCs were resuspended at 1% HCT in 0.9% NaCl, and treated either with 0, 0.001, 0.0025, 0.005 or 0.01% H_2_O_2_ to generate oxidative stress. After 10 min at room temperature, the emitted fluorescence was quantified by flow cytometry (BD FACSVia and BD FACSVia Research Software, BD Biosciences, San Jose, CA, USA). For the analysis, the fluorescence emitted at different oxidant concentrations was reported and the area under the curve (AUC, with a baseline corresponding to the value of fluorescence at 0% H_2_O_2_) was calculated using PRISM software.

### 4.7. Quantification of Extracellular-Glucose and Lactate, and Intracellular-2,3-DPG and ATP Levels

The extracellular concentrations of glucose and lactate were measured in the supernatants (stored at −80 °C), using the BioChain Glucose Assay (kit Z5030025, Newark, CA, USA) and the BioVision Lactate Colorimetric Assay (kit II, K627-100). Prior to (intracellular) ATP and 2,3-DPG quantification, the RBC samples (stored at −80 °C) were deproteinized with the BioVision Deproteinizing Sample Preparation Kit (K808-200, Milpitas, CA, USA) or according to the method described in the 2,3-Diphosphoglycerate assay (kit 10148334001, Mannheim, Germany) from Roche.The intracellular ATP and 2,3-DPG levels were quantified using the ATPlite Luminescence ATP Detection Assay System (kit 6016941, Waltham, MA, USA) from PerkinElmer and the kit from Roche, respectively.

### 4.8. Metabolomic Analysis

A panel of intracellular metabolites was quantified in the UA-AA-treated and control RBCs after 2, 8, 15, 29 and 43 days of storage. The extraction of the intracellular RBC metabolites consisted in three successive steps. First, 450 µL of cold methanol (−28 °C) were added on the frozen pellets of washed packed RBCs (150 µL). The tubes were carefully vortexed. The samples were kept 5 min on ice before being sonicated in an ice bath during 10 min. The vortex and sonication steps were repeated twice. The tubes were then centrifuged during 15 min at 4 °C and 21,000× *g*. The supernatant was put aside and kept on ice, the bottom part which formed a solid aggregate was further processed. For the second extraction step, 225 µL of 50% iced methanol (−28 °C) was added on the aggregate. The sample was vortexed, sonicated and centrifuged (as before), and the supernatant was added to the first one. Finally, for the third and last step, 225 µL of cold dH_2_O (purity of 18.2 MΩ.cm) at 4 °C was added on the aggregate, the process was then similar as for the second step. The extracts obtained from the three successive steps were stored at −80 °C prior analysis.

Before the analysis, the extracts were filtered through 0.2 µm filters. The quantification was done according to the method described by Ghorbaniaghdam et al. [50]. Briefly, the metabolites were separated by ultra-performance liquid chromatography (UPLC) and analyzed by tandem mass spectrometry (MS/MS). To achieve accurate and sensitive quantification, a multiple reaction monitoring (MRM) approach was applied. The resulting AUC (elution time vs. MRM signal) was calculated and compared to calibrations curves obtained with commercial standards for each series of analysis.

### 4.9. Data Presentation and Statistical Analyses

Prism 8 for macOS (GraphPad Software Inc., San Diego, CA, USA), version 8.4.2 (464) was used for data presentation and statistical analyses. Multiple comparisons were done to evaluate the effect of the treatment using 2way ANOVA.

## 5. Conclusions

Addition of protective molecules and/or modifications of the additive solution composition used for the storage of the labile blood products is probably one of the most convenient and cost-effective approaches to guarantee a product of good quality for all patients. Moreover, the use of compounds that are naturally found in the human organism, drastically reduces the risks of adverse reactions. The aim of the present research was to investigate if UA and AA supplementation could prevent or limit the lesions associated with the storage of RBCs in blood bags. The concentrations were chosen in the aim of replenishing physiological levels of these two endogenous molecules.

It was observed that the correction of plasma dilution by UA and AA addition increased the global antioxidant power and triggered a direct modification of the level of several intracellular metabolites that persisted through the storage. However, it seems that the effects of the treatment were not strong enough to prevent the occurrence of all types of storage lesions.

In future in vitro experiments, simulation of transfusion via post-storage incubation at 37 °C in plasma could be used to better reflect the behavior of the cells after transfusion [51,52]. It could also be interesting to test higher concentrations of antioxidants. In addition, the supplementation should be done immediately after whole blood processing, which was not possible in this study because of logistic issues. Finally, other metabolites than urate are probably influenced by the plasma dilution and might be considered too.

Beyond transfusion medicine issues, the present observations gain insight and open the discussion around the impact of storage on RBC metabolism.

## Abbreviations

The following abbreviations are used in this manuscript: 2,3-diphosphoglycerate (2,3-DPG), ascorbic acid (AA), aldolase (ALD), adenosine mono-/di-/tri-phosphate (AMP/ADP/ATP), antioxidant power (AOP), area under the curve (AUC), citrate-phosphate-dextrose (CPD), cysteine (Cys), 2′,7′-dichlorofluorescin diacetate (DCFH-DA), digital holographic microscopy (DHM), Ferric Reducing Ability of Plasma (FRAP), glucose-6-phosphate dehydrogenase (G6PD), glyceraldehyde 3-phosphate dehydrogenase (GADPH), glutamate (Glu), glycine (Gly), guanosine mono-/di-/tri-phosphate (GMP/GDP/GTP), reduced/oxidized glutathione (GSH/GSSG), hydrogen peroxide (H_2_O_2_), haematocrit (HCT), hexokinase (HK), inosine monophosphate (IMP), lactate dehydrogenase (LDH), mean corpuscular volume (MCV), malate dehydrogenase (MDH), multiple reaction monitoring (MRM), mass spectrometry (MS), nicotinamide adenine dinucleotide (reduced) (NAD(H)), nicotinamide adenine dinucleotide phosphate (reduced) (NADP(H)), phosphofructokinase (PFK), phosphoglucoisomerase (PGI), inorganic phosphate/pyrophosphate (Pi/PPi), pyruvate kinase (PK), pentose phosphate pathway (PPP), phosphoribosyl diphosphate (PRPP), red blood cell (RBC), red blood cell concentrate (RCC), reactive oxygen species (ROS), saline-adenine-glucose-mannitol (SAGM), S-adenosylmethionine cycle (SAM), standard deviation of the optical path difference (SD-OPD), standard deviation of the red blood cell distribution width (SD-RDW), tricarboxylic acid cycle (or Krebs cycle) (TCA), tetrahydrofolate (THF), uric acid (UA), ultra-performance liquid chromatography (UPLC), xanthosine monophosphate (XMP) and xanthine oxidase (XO).

## Appendix B

**Table A1:** Donor’s characteristics. Sex, age and blood group (ABO and Rhesus D) of the donor.

Sample	Sex	Age/Year	Blood Group
RCC 1	Male	52	A +
RCC 2	Male	54	A +
RCC 3	Male	27	B +
RCC 4	Male	61	A +

RCC: red blood cell concentrate.
